# Gender differences in prevalence and associations between cognitive symptoms and suicidal ideation in patients with recurrent major depressive disorder: findings from the Chinese NSSD study

**DOI:** 10.1186/s12888-024-05557-x

**Published:** 2024-01-31

**Authors:** Ruizhi Mao, Chenglei Wang, Lvchun Cui, David Mellor, Zhiguo Wu, Yiru Fang

**Affiliations:** 1 Clinical Research Center and Division of Mood Disorders, Shanghai Mental Health Center, Shanghai Jiao Tong University School of Medicine, Shanghai, China; 2 Shanghai Changning District Mental Health Center, Shanghai, China; 3 School of Psychology, Deakin University, Melbourne, Australia; 4 Department of Psychological Medicine, Renji Hospital, Shanghai Jiao Tong University School of Medicine, 160 Pujian Road, Shanghai, 200127 China; 5 Shanghai Yangpu District Mental Health Center, Shanghai University of Medicine & Health Sciences, Shanghai, China; 6 Department of Psychiatry & Affective Disorders Center, Ruijin Hospital, Shanghai Jiao Tong University School of Medicine, 197 Ruijin 2nd Road, Shanghai, 200025 China; 7 CAS Center for Excellence in Brain Science and Intelligence Technology, Shanghai, China; 8 Shanghai Key Laboratory of Psychotic Disorders, Shanghai, China

**Keywords:** Major depressive disorder, Recurrent depression, Gender differences, Suicidal ideation, Cognitive symptoms

## Abstract

**Background:**

This study aimed to explore gender differences in associations between cognitive symptoms and suicidal ideation (SI) among patients with recurrent major depressive disorder (MDD).

**Methods:**

We recruited 1222 patients with recurrent MDD from the National Survey on Symptomatology of Depression (NSSD), a survey designed to investigate the symptoms experienced during current major depressive episodes in China. A four-point Likert questionnaire was used to assess the frequency of cognitive symptoms and SI in the past two weeks.

**Results:**

Gender differences in clinical features and cognitive symptoms of participants with recurrent MDD were found. Specifically, male patients had a higher prevalence of memory loss, decreased verbal output, indecisiveness, and impaired interpersonal relationships, while female patients exhibited a higher prevalence of impaired social and occupational functioning (all *P* < 0.05). No significant difference in SI prevalence was found between male and female patients. The logistic regression analysis revealed that in male patients, SI was associated with indecisiveness and impaired interpersonal relationships. In female patients, reduced verbal output and impaired social and professional functions were also associated with SI in addition to the above-mentioned variables.

**Conclusion:**

The findings of gender differences in associations between cognitive symptoms and SI highlight the need to carefully assess gender-specific cognitive predictors of SI in patients with recurrent MDD. This has further implications for more targeted prevention and treatment strategies for SI based on gender.

**Supplementary Information:**

The online version contains supplementary material available at 10.1186/s12888-024-05557-x.

## Background

Major depressive disorder (MDD) is a recurrent mental illness. According to the findings of the Sequenced Treatment Alternatives to Relieve Depression (STAR*D) study, 74% of participants with MDD experienced recurrences, with 18% having 10 or more lifetime major depressive episodes [1]. This highlights the need to manage recurrent depression in clinical practice. Several studies have suggested that gender differences exist in the prevalence, clinical symptomatology, treatment outcomes, and the tendency for recurrence in patients with MDD. In comparison to male patients with MDD, female patients are more susceptible to relapse and experience longer periods of major depressive episodes [2–4].

Moreover, it is of note that suicidal ideation (SI) has been reported to be linked with a higher risk of recurrence in female patients with MDD than in male patients [3]. SI should be considered a warning sign requiring clinical attention since some patients with such thoughts could progress to suicidal behavior [5]. Thus, exploration of gender differences in SI among patients with recurrent depression might help develop screening, intervention, and prevention strategies for suicidality in patients with MDD.

In addition to emotional symptoms, cognitive dysfunction is a core trait of MDD, including areas such as memory, attention, processing speed, and executive function [6, 7]. However, there appear to be cognitive impairment differences between male and female MDD patients [8]. Research has found that first-diagnosed drug-naïve depressed female patients exhibit more severe cognitive impairment than male patients in the visuospatial and constructional domains [9]. On the other hand, male patients do not perform as well as female patients in information processing speed [10]. Distinct cognitive domains show gender-specific brain activation patterns, suggesting that males and females recruit different neural networks when performing the same cognitive tasks [11–13]. Sex hormones, inflammation, and stress may also contribute to these different cognitive performances [14, 15]. For example, studies have shown that estradiol and testosterone mediate resting-state functional connectivity in females [16, 17].

Compared to first-episode patients, those with recurrent depressive episodes appear to experience more severe cognitive impairments, which have been identified as a significant predictor of relapse [18, 19]. However, currently, there is a lack of systematic research examining gender differences in the clinical correlates of cognitive symptoms among MDD individuals with recurrent depression. One area that is worthy of examination is suicidal ideation and behaviors. Pu et al. [20] found that executive function, motor speed, and overall neuropsychological performance are linked to SI in individuals diagnosed with MDD. A study conducted among depressed women in Iran found that those who had SI or a history of suicide attempts had poorer cognitive control and cognitive emotion regulation abilities. Cognitive control deficits indirectly impact suicidal behavior through cognitive emotion regulation strategies such as self-blame, acceptance, rumination, and catastrophizing [21]. Furthermore, cognitive improvements such as increased processing speed are associated with a reduction in SI following antidepressant treatment [22]. These findings highlight the importance of targeting cognitive symptoms to prevent suicidal behaviors in individuals with depression. Further, examining the impact of gender differences in SI, cognitive symptoms, and their relationship is crucial for MDD individuals with recurrent depression, who are known to be at higher risk for suicide.

In this study, we aim to investigate the potential gender differences in the following aspects among Chinese patients with recurrent MDD: (1) clinical characteristics, (2) the prevalence of cognitive symptoms and SI, and (3) associations between SI and cognitive symptoms.

## Material and methods

### Participants

The 1222 patients with recurrent MDD were drawn from the National Survey on Symptomatology of Depression (NSSD). The NSSD consecutively recruited 3275 patients with MDD from outpatient or inpatient populations in the psychiatric settings of 16 general hospitals and 16 mental health centers in 22 different cities across 15 provinces. The inclusion criteria were as follows: (a) aged ≥ 18 years; (b) met the Diagnostic and Statistical Manual of Mental Disorders (DSM-IV) TR criteria for MDD (current episode). The exclusion criteria were: (a) a lifetime diagnosis of bipolar disorder, psychotic disorders, or mental retardation; (b) female patients who were pregnant or breastfeeding; (c) risk of imminent suicide, specifically, plans to take any active steps to prepare for a suicide attempt in which the patient intended to kill himself/herself in the next hours, as judged by research psychiatrists, or any signs of homicide; (d) having undergone electroconvulsive therapy within 1 month before entering the study.

### Study procedures and measures of cognitive symptoms and SI

The NSSD aimed to investigate the magnitude of symptoms associated with current major depressive episodes in MDD patients, both within and beyond the DSM framework, in real-world clinical settings across China. We developed a doctor-rating questionnaire with 64 items, covering emotional, cognitive, atypical, somatic, psychotic, and other symptoms of MDD. Participants were requested to report the frequency of each symptom experienced over the past two weeks. The scale ranged from 0 to 3, with 0 indicating "not at all", 1 indicating "several days", 2 indicating "more than half the days", and 3 indicating "nearly every day".

The questionnaire was designed based on Chinese versions of materials, including (a) diagnostic manuals such as International Classification of Diseases, 10th Edition (ICD-10), DSM-IV/DSM-5 and the Chinese Classification of Mental Disorders, 3rd edition (CCMD-3); (b) standard rating scales with proven reliability and validity among Chinese populations, including the Montgomery-Asberg Depression Rating Scale, the Hamilton Rating Scale for Depression/Anxiety, the Self-Rating Depression/Anxiety Scale, the Quick Inventory of Depressive Symptomatology, and the Patient Health Questionnaires; (c) insights from top experts on crucial signs of depression found in Chinese textbooks and literature on MDD clinical presentations. The questionnaire and study protocol were discussed and achieved consensus within the research team and a consultant group comprising 10 senior psychiatrists with advanced expertise in clinical work and depression research in China. The raters, all psychiatrists with a minimum of 5 years of practice, underwent thorough training for this project. The inter-investigator correlation coefficient exceeded 0.8 for each interviewer, indicating the questionnaire's good reliability.

Among these 64 symptoms, cognitive symptoms included the following items: (a) memory loss, (b) decrease in verbal, (c) poor concentration, (d) indecisiveness, (e) thinking retardation, (f) social withdrawal, (g) poor interpersonal relationships, (h) impaired social and professional functions [19]. We used a low threshold (score ≥ 1) to identify the presence of SI and emphasize the significance of evaluating the risk of suicide [23].

Approval was obtained from the Institutional Review Board of Shanghai Mental Health Center (IRB00002733-Shanghai Mental Health Center, China), and all patients provided written informed consent before the study entry.

### Statistical analyses

Statistical analyses were conducted using SPSS version 26.0 (SPSS Inc., Chicago, IL, USA), and a significance level of *P* < 0.05 (two-tailed) was used. The homogeneity of variance was evaluated using Levene's test, and the normality of data distribution for each group was assessed using the Kolmogorov–Smirnov test. Continuous variables were analyzed using Student’s *t*-test, while categorical variables were compared using the chi-squared test. Non-normally distributed variables were analyzed using the Mann–Whitney U test. Spearman correlation coefficients were calculated separately for female and male patients to examine the associations between cognitive symptoms and SI. Furthermore, a logistic regression analysis was performed to determine the cognitive variables significantly associated with SI while controlling for age, education level, number of depressive episodes, duration of current episode, total course of depression, use of mood stabilizers, smoking habits, and alcohol consumption as potential covariates. To correct for multiple tests, Bonferroni corrections were applied.

## Results

### Gender differences in sociodemographic factors and clinical characteristics of the participants

As shown in Table 1, a total of 1222 MDD patients with recurrent depression (476 males and 746 females) were included in this study. Compared to male patients, female patients exhibited an older age, a lower proportion of individuals with postgraduate and above-level education, lesser employment rates, and lower rates of tobacco and alcohol use in the analysis of sociodemographic factors (all *P* < 0.05). In terms of clinical features, female patients exhibited a significantly shorter total course of depression (*P* = 0.026) and duration of the current episode (*P* = 0.000) compared to male patients. The medication profiles of patients are presented in Table 2, revealing that, except for a higher usage rate of mood stabilizers among male patients with recurrent depression, there were no gender differences in the utilization of other medications.

**Table 1:** Demographic and clinical characteristics of male and female MDD patients with recurrent depression

Variables	Total (*n* = 1222)	Male (*n* = 476)	Female (*n* = 746)	*Z/t/x*^*2*^	*P*
Demographic characteristics					
Age (years)	44.48 ± 13.98	43.32 ± 13.77	45.22 ± 14.07	-2.317	0.021^*^
Education level					
Below undergraduate	754 (61.7)	286 (62.0)	486 (66.6)	6.592	0.037^*^
Undergraduate and above	382 (31.3)	158 (34.3)	224 (31.9)		
Postgraduate and above	28 (2.3)	17 (3.7)^*^	11 (1.6)^*^		
Marital status					
Unmarried	197 (16.1)	91 (19.4)	106 (14.4)	5.260	0.154
Married	933 (76.4)	350 (74.5)	583 (79.0)		
Divorced	43 (3.5)	16 (3.4)	27 (3.7)		
Widowed	35 (2.9)	13 (2.8)	22 (3.1)		
Employment status					
Unemployed	307 (25.1)	95 (20.7)^*^	212 (29.6)^*^	31.102	0.000^**^
Employed	577 (47.2)	272 (59.3)^*^	305 (42.6)^*^		
Student	53 (4.3)	17 (3.7)	36 (5.0)		
Retired	238 (19.5)	75 (16.3)^*^	163 (22.8)^*^		
Smoking habits					
Non-smoker	998 (81.7)	294 (61.9)^*^	704 (94.6)^*^	209.264	0.000^**^
Ex-smoker	109 (8.9)	90 (18.9)^*^	19 (2.6)^*^		
Current smoker	112 (9.2)	91 (19.2)^*^	21 (2.8)^*^		
Alcohol consumption					
Non-drinker	1022 (83.6)	319 (67.4)^*^	703 (94.6)^*^	159.364	0.000^**^
Ex-drinker	119 (9.7)	93 (19.7)^*^	26 (3.5)^*^		
Current drinker	75 (6.1)	61 (12.9)^*^	14 (1.9)^*^		
Clinical characteristics					
Total course of depression (months)	25 (6, 60)	28.5 (7, 66)	24 (5.69, 56)	-2.223	0.026^*^
Duration of current episode (months)	2 (1, 4.5)	2.25 (1, 5.44)	2 (1, 4)	-3.547	0.000^**^
Number of depressive episodes	2 (2, 3)	3 (2, 3)	2 (2, 3)	-1.029	0.303
Number of hospitalization	0 (0, 2)	0 (0, 2)	0 (0, 2)	-0.382	0.702
Antidepressants usage during first-onset	1113 (91.1)	437 (39.3)	676 (60.7)	0.510	0.475
Medical history of physical illnesses	318 (26.1)	121 (25.5)	197 (26.5)	0.136	0.713
Comorbid hypertension	108 (8.8)	50 (10.5)	58 (7.8)	2.687	0.101
Comorbid diabetes	44 (3.6)	15 (3.2)	29 (3.9)	0.454	0.501

Data were presented as Mean ± S.D., number plus rate (n, %), and median (P25–P75)

^*^ *P* < 0.05

^**^ *P* < 0.0

**Table 2:** Medication treatment of male and female MDD patients with recurrent depression

Medication	Total (*n* = 1222)	Male (*n* = 476)	Female (*n* = 746)	*x*^*2*^	*P*
SSRIs	819 (67.1)	332 (69.7)	487 (65.4)	2.522	0.112
SNRIs	196 (16.1)	74 (15.5)	122 (16.4)	0.148	0.700
TCAs	64 (5.2)	31 (6.5)	33 (4.4)	2.537	0.111
NaSSAs	74 (6.1)	29 (6.1)	45 (6.0)	0.001	0.970
NDRIs	3 (0.2)	1 (0.2)	2 (0.3)	0.040	0.841
SARIs	24 (2.0)	5 (1.1)	19 (2.6)	3.391	0.066
Mood stabilizer	103 (8.4)	51 (10.7)	52 (7.0)	5.244	0.022^*^
Antipsychotics	242 (19.8)	89 (18.7)	153 (20.5)	0.711	0.701
Benzodiazepines	646 (52.9)	247 (51.9)	399 (53.6)	0.324	0.569

Data were presented as number plus rate (n, %). SSRIs, selective serotonin reuptake inhibitors. SNRIs, serotonin-norepinephrine reuptake inhibitors. TCAs, tricyclic antidepressants. NaSSAs, noradrenergic and specific serotonergic antidepressants. NDRIs, norepinephrine-dopamine reuptake inhibitors. SARIs, serotonin antagonist and reuptake inhibitors

^*^ *P* < 0.05

### Gender differences in the prevalence of cognitive symptoms and SI

**Fig. 1:**
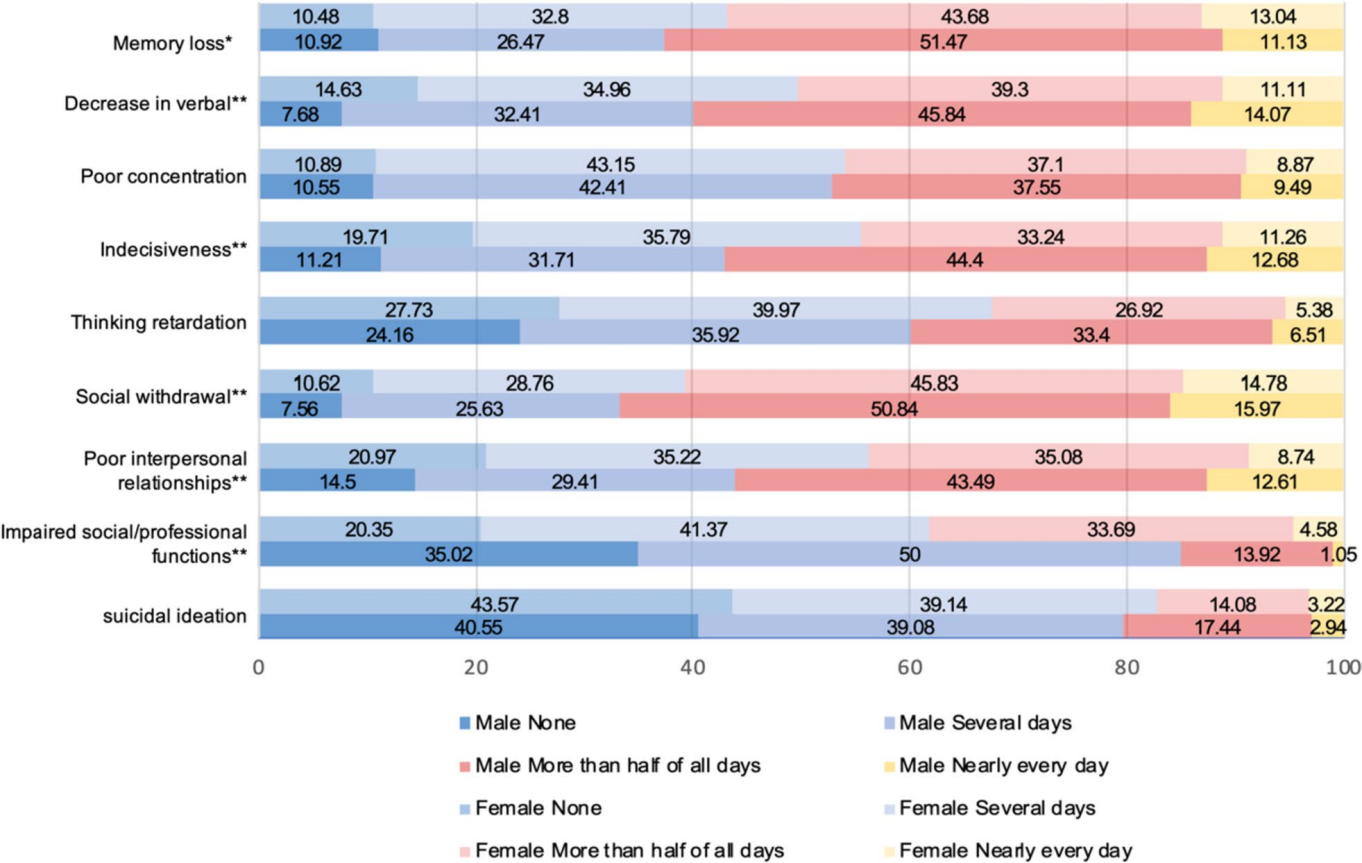
Prevalence of cognitive symptoms and SI between male and female patients with recurrent MDD. ^*^: *P* < 0.05, ^**^: *P* < 0.01; SI: suicidal ideation, MDD: major depressive disorder

Significant gender differences were found in the frequency of cognitive symptoms, including memory loss, decrease in verbal activity, indecisiveness, poor interpersonal relationships, and impaired social and professional functioning (all *P* < 0.05). However, there was no significant difference in the prevalence of SI between male and female patients (Fig. 1 and Table S1).

For “memory loss”, 32.8% of female patients reported experiencing this symptom for "several days" in the past two weeks, compared to only 26.47% of male patients. However, 51.47% of male participants reported experiencing this symptom for "more than half of all days", a higher prevalence than that of 43.68% of female patients (*P* < 0.05). For "decrease in verbal" and "indecisiveness", male participants had a higher prevalence of experiencing these symptoms "more than half of the day" compared to females (45.84% vs 39.30%, 44.40% vs 33.24%, all *P* < 0.05). Compared to male patients, more females did not have the symptom of "poor interpersonal relationships" or only had it for "several days" (14.50% vs 20.97%, 29.41% vs 35.22%, all *P* < 0.05). Meanwhile, 43.49% and 12.61% of male patients reported being significantly bothered by this symptom for "more than half of all days" or "nearly every day", with a higher prevalence than female patients (35.08%, 8.74%). Female patients with recurrent depression had a more severe impact on their social and professional functions. 33.69% of female patients felt significantly affected by it for more than half the time, while 4.58% of females experienced it almost every day (all *P* < 0.05).

### Gender differences in the associations of cognitive symptoms and SI

**Fig. 2:**
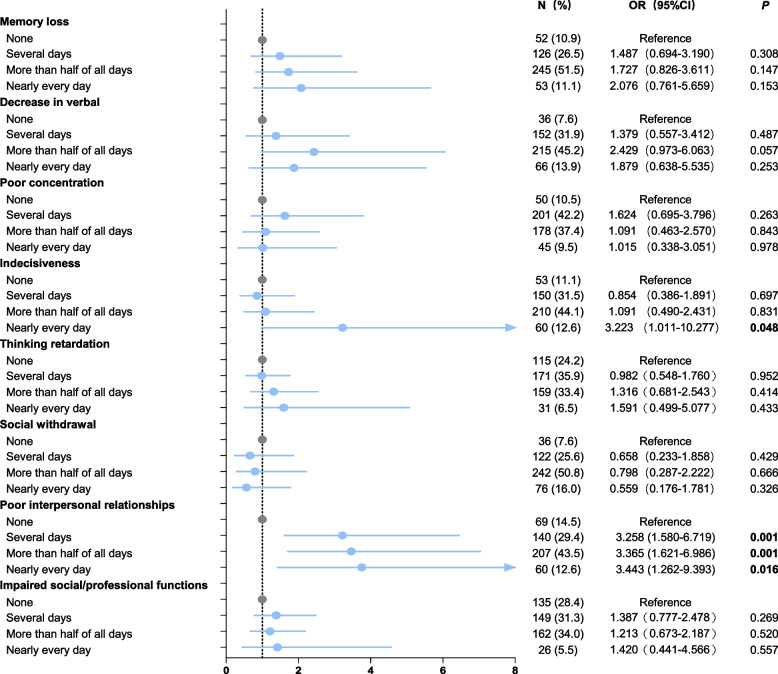
The associations between cognitive symptoms and SI in male patients with recurrent MDD. SI: suicidal ideation, MDD: major depressive disorder

**Fig. 3:**
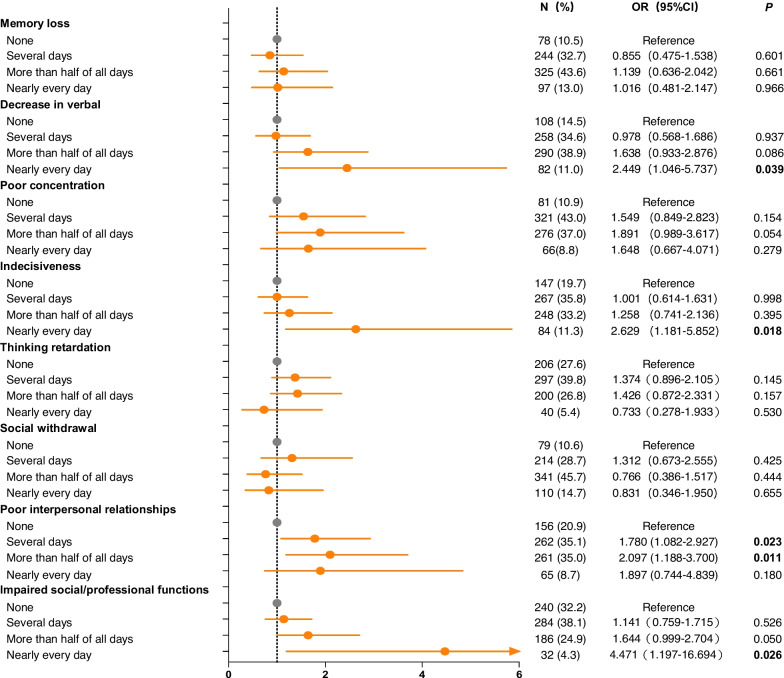
The associations between cognitive symptoms and SI in female patients with recurrent MDD. SI: suicidal ideation, MDD: major depressive disorder

Table S2 presented the results of the Spearman correlation analysis, demonstrating significant positive correlations between frequencies of cognitive symptoms and SI in both male and female patients with recurrent depression (all *P* < 0.01). All these associations between cognitive symptoms and SI remained significant even after Bonferroni correction (α = 0.05/8 = 0.0063). Logistic regression analysis was conducted based on the presence of SI, controlling for potential confounding variables such as age, education level, number of depressive episodes, duration of current episode, total course of depression, use of mood stabilizers, smoking habits, and alcohol consumption. As depicted in Fig. 2, male patients with recurrent MDD who experienced "indecisiveness" almost daily (OR = 3.223, 95% CI: 1.011—10.277) exhibited an increased risk of SI. Similarly, male participants who reported “poor interpersonal relationships” for several days (OR = 3.258, 95% CI: 1.580—6.719), more than half of all days (OR = 3.365; 95% CI: 1.621—6.986), or nearly every day (OR = 3.443, 95% CI: 1.262—9.393) had a higher likelihood of experiencing SI compared to those without this symptom. Figure 3 depicts the associations between cognitive symptoms and SI in female patients. Individuals who experienced a "decrease in verbal," "indecisiveness," and "impaired social and professional functions" on an almost daily basis (OR = 2.449, 95% CI: 1.046—5.737; OR = 2.629, 95% CI: 1.181—5.852; OR = 4.471, 95% CI: 1.197—16.694) had an increased risk of SI when compared to those without these symptoms. Moreover, female participants who experienced “poor interpersonal relationships” for several days (OR = 1.780, 95% CI: 1.082—2.927) or more than half of all days (OR = 2.097, 95% CI: 1.188—3.700) also exhibited a higher risk of SI compared to those without this symptom.

## Discussion

The present study identified three main findings in patients with recurrent MDD. Firstly, gender differences were observed in the sociodemographic and clinical characteristics. Secondly, male patients exhibit a higher prevalence of certain cognitive symptoms, while there is no significant difference in the prevalence of SI between males and females. Thirdly, female patients with recurrent depression have a broader range of cognitive symptoms that are associated with SI compared to male patients. These symptoms included a decrease in verbal activity, indecisiveness, impaired social relationships, and impaired social and professional functioning.

### Gender differences in sociodemographic and clinical features

In this study, female patients exhibited older age, lower education level, and lower proportion of employment. This suggests potential limitations in accessing social support networks and the knowledge necessary for effectively managing related symptoms and preventing relapse [24, 25]. Interestingly, we found that female patients had a shorter duration of the current episode and total illness course compared to the males, contradicting earlier studies [26]. This discrepancy may be due to our recruitment from outpatient or inpatient settings, where female patients may have sought help earlier due to lower tolerance for certain factors like somatic symptoms [27].

### Gender differences in the prevalence of cognitive symptoms and SI

Male patients with recurrent depression exhibited more severe cognitive symptoms shown by significantly higher frequencies of memory loss, slowed thinking, indecisiveness, and decreased verbal expression compared to females, all occurring more than half of the time. This may be attributed to a tendency of male patients with MDD to evaluate themselves based on behaviors like psychomotor functioning and cognitive impairments, while female patients are more likely to rely on feelings to assess their depression [28]. Male patients also showed a higher frequency of impaired interpersonal relationships. Previous research also suggests that male depressive patients tend to utilize avoidance coping strategies, such as communication refusal and distancing from others [29]. Of course, we cannot overlook the impact of lifestyle habits on cognitive function in male patients. In this study, the proportion of male patients who smoke and consume alcohol is higher than that of females, and research findings are associating both factors with cognitive decline. Specifically, alcohol use is particularly evident in impairing verbal memory and learning [30, 31]. Furthermore, the impact of more frequent mood stabilizer use on the cognitive function of male patients also needs to be further explored in future studies, along with the consideration of tobacco and alcohol use [32].

Despite the higher prevalence of cognitive symptoms in male patients, their impact on social and occupational functioning is not as severe as in females, suggesting the involvement of other factors. Women often report additional symptoms such as insomnia, low appetite, and physical complaints, which can adversely affect their daily quality of life [33]. Our study found no gender differences in the prevalence of SI, which aligns with inconsistent previous findings. Fang et al. identified male gender as a risk factor for SI, while other studies reported higher rates among females [34–36]. This discrepancy may be due to our study's focus on individuals with recurrent depression. Importantly, successful prevention of the recurrence of MDD could potentially lead to a significant reduction in the occurrence or reoccurrence of SI [37].

### Gender differences in the associations of cognitive symptoms and SI

Our analysis of male and female patients found that indecisiveness and impaired social relationships were significantly associated with SI. Indecisiveness reflects compromised decision-making ability in individuals with depression. Understanding the underlying factors contributing to indecisiveness is crucial. Neurobiological studies have revealed altered brain activity in decision-making regions among adults with a history of suicidal behaviors [38]. Psychological factors such as negative self-perception, self-doubt, and rumination can further exacerbate indecisiveness and its link to SI [39, 40]. Impaired interpersonal relationships also showed a significant correlation with SI, consistent with the interpersonal theory of suicide which suggests that persistent SI is often accompanied by unmet interpersonal needs, including high levels of thwarted belongingness (feeling like a burden to others) and perceived burdensomeness (feeling alienated from others) [41, 42]. Addressing these cognitive-affective states, Interpersonal Psychotherapy (IPT) has shown effectiveness in improving social functioning, reducing interpersonal stressors, and decreasing SI [43].

In addition to the aforementioned cognitive symptoms, this study found that SI in female patients is associated with more other cognitive symptoms than in male patients, including reduced verbal output and impaired social and professional functioning. Reduced speech can limit interaction and communication, affecting social support networks. Self-disclosure is essential for healthy adaptation and has been effective in reducing complicated grief [44]. This partially explains the significant correlation between decreased verbal output and SI. Thus, careful evaluation of interpersonal risk factors is clinically useful to identify early indicators of SI and develop intervention strategies for SI and self-harming behaviors [45]. The relationship between impaired social and professional functions and SI reminds clinicians that the treatment targets for MDD should shift from symptom control to achieving functional recovery [46]. The aforementioned viewpoint is also in line with our findings in female patients with recurrent depression.

### Limitations

There are some limitations in this study. Firstly, it is a cross-sectional study, and we cannot determine the causal relationships between cognitive symptoms and SI. The current findings should be regarded as preliminary and require confirmation through longitudinal studies. Secondly, this study utilized a non-validated questionnaire to assess each symptom that occurred in patients. The use of standard assessment tools might be helpful to more accurately assess cognitive functions and SI in future research. Finally, potential confounding effects of psychiatric comorbidities or specific medical comorbidities could not be ruled out.

## Conclusion

In conclusion, we found gender differences in the associations between cognitive symptoms and suicidal ideation, which highlights the need to cautiously assess gender-specific cognitive predictors of SI among patients with recurrent MDD. This has further implications for more targeted prevention and treatment strategies for suicide on a gender basis. Targeting these cognitive symptoms in treatment or training may also contribute to better patient recovery.

### Supplementary Information

**Additional file 1: Table S1.** Prevalence of cognitive symptoms and suicidal ideation between male and female patients. **Table S2.** Correlation between suicidal ideation and cognitive symptoms in male and female patients with recurrent MDD.

## Data Availability

No datasets were generated or analysed during the current study.

## Abbreviations

SI: Suicidal ideation
MDD: Major depressive disorder
NSSD: National Survey on Symptomatology of Depression
STAR*D: Sequenced Treatment Alternatives to Relieve Depression
ICD-10: International Classification of Diseases, 10th Edition
CCMD-3: Chinese Classification of Mental Disorders, 3rd edition
DSM: Diagnostic and Statistical Manual of Mental Disorders
OR: Odds ratio
SSRIs: Selective serotonin reuptake inhibitors
SNRIs: Serotonin-norepinephrine reuptake inhibitors
TCAs: Tricyclic antidepressants
NaSSAs: Noradrenergic and specific serotonergic antidepressants
NDRIs: Norepinephrine-dopamine reuptake inhibitors
SARIs: Serotonin antagonists and reuptake inhibitors
