# Supplementary material for: Gender differences in prevalence and associations between cognitive symptoms and suicidal ideation in patients with recurrent major depressive disorder: findings from the Chinese NSSD study
Source: BMC Psychiatry. 2024 Jan 31;24:83. doi: 10.1186/s12888-024-05557-x (PMC10829390; doi:10.1186/s12888-024-05557-x)
Supplement: Supplementary file 1 — Additional file 1: Table S1. Prevalence of cognitive symptoms and suicidal ideation between male and female patients. Table S2. Correlation between suicidal ideation and cognitive symptoms in male and female patients with recurrent MDD. [file 12888_2024_5557_MOESM1_ESM.docx]

**Table S1**

Prevalence of cognitive symptoms and suicidal ideation between male and female patients.

| Variables |  | Gender (%) | | | χ^2^ | *P* | *P* for trend |
| --- | --- | --- | --- | --- | --- | --- | --- |
|  |  | Male |  | Female |  |  |  |
| Memory loss | None | 52 (10.92) |  | 78 (10.48) | 8.505 | 0.037^*^ | 0.470 |
|  | Several days | 126 (26.47) ^*^ |  | 244 (32.80) ^*^ |  |  |  |
|  | More than half of all days | 245 (51.47) ^*^ |  | 325 (43.68) ^*^ |  |  |  |
|  | Nearly every day | 53 (11.13) |  | 97 (13.04) |  |  |  |
| Decrease in verbal | ­ None | 36 (7.68) ^*^ |  | 108 (14.63) ^*^ | 17.175 | 0.001^**^ | 0.000^**^ |
|  | Several days | 152 (32.41) |  | 258 (34.96) |  |  |  |
|  | More than half of all days | 215 (45.84) ^*^ |  | 290 (39.30) ^*^ |  |  |  |
|  | Nearly every day | 66 (14.07) |  | 82 (11.11) |  |  |  |
| Poor concentration | None | 50 (10.55) |  | 81 (10.89) | 0.207 | 0.976 | 0.666 |
|  | Several days | 201 (42.41) |  | 321 (43.15) |  |  |  |
|  | More than half of all days | 178 (37.55) |  | 276 (37.10) |  |  |  |
|  | Nearly every day | 45 (9.49) |  | 66 (8.87) |  |  |  |
| Indecisiveness | None | 53 (11.21) ^*^ |  | 147 (19.71) ^*^ | 24.236 | 0.000^**^ | 0.000^**^ |
|  | Several days | 150 (31.71) |  | 267 (35.79) |  |  |  |
|  | More than half of all days | 210 (44.40) ^*^ |  | 248 (33.24) ^*^ |  |  |  |
|  | Nearly every day | 60 (12.68) |  | 84 (11.26) |  |  |  |
| Thinking retardation | None | 115 (24.16) |  | 206 (27.73) | 7.418 | 0.060 | 0.017^*^ |
|  | Several days | 171 (35.92) |  | 297 (39.97) |  |  |  |
|  | More than half of all days | 159 (33.40) ^*^ |  | 200 (26.92) ^*^ |  |  |  |
|  | Nearly every day | 31 (6.51) |  | 40 (5.38) |  |  |  |
| Social withdrawal | None | 36 (7.56) |  | 79 (10.62) | 5.698 | 0.127 | 0.035^*^ |
|  | Several days | 122 (25.63) |  | 214 (28.76) |  |  |  |
|  | More than half of all days | 242 (50.84) |  | 341 (45.83) |  |  |  |
|  | Nearly every day | 76 (15.97) |  | 110 (14.78) |  |  |  |
| Poor interpersonal relationships | None | 69 (14.50) ^*^ |  | 156 (20.97) ^*^ | 19.147 | 0.000^**^ | 0.000^**^ |
|  | Several days | 140 (29.41) ^*^ |  | 262 (35.22) ^*^ |  |  |  |
|  | More than half of all days | 207 (43.49) ^*^ |  | 261 (35.08) ^*^ |  |  |  |
|  | Nearly every day | 60 (12.61) ^*^ |  | 65 (8.74) ^*^ |  |  |  |
| Impaired social and professional functions | None | 166 (35.02) ^*^ |  | 151 (20.35) ^*^ | 83.406 | 0.000^**^ | 0.000^**^ |
|  | Several days | 237 (50.00) ^*^ |  | 307 (41.37) ^*^ |  |  |  |
|  | More than half of all days | 66 (13.92) ^*^ |  | 250 (33.69) ^*^ |  |  |  |
|  | Nearly every day | 5 (1.05) ^*^ |  | 34 (4.58) ^*^ |  |  |  |
| Suicidal ideation | None | 193 (40.55) |  | 325 (43.57) | 2.831 | 0.418 | 0.221 |
|  | Several days | 186 (39.08) |  | 292 (39.14) |  |  |  |
|  | More than half of all days | 83 (17.44) |  | 105 (14.08) |  |  |  |
|  | Nearly every day | 14 (2.94) |  | 24 (3.22) |  |  |  |

Note: Data were presented as number plus rate (n, %).

* *P* < 0.05, ** *P* < 0.01

**Table S2**

Correlation between suicidal ideation and cognitive symptoms in male and female patients with recurrent MDD.

| Variables | Total  (n = 1222) | |  | Male  (n = 476) | |  | Female  (n = 746) | |
| --- | --- | --- | --- | --- | --- | --- | --- | --- |
|  | r | *P* |  | r | *P* |  | r | *P* |
| Memory loss | 0.145 | 0.000^**^ |  | 0.176 | 0.001^**^ |  | 0.125 | 0.001^**^ |
| Decrease in verbal | 0.232 | 0.000^**^ |  | 0.207 | 0.000^**^ |  | 0.241 | 0.000^**^ |
| Poor concentration | 0.190 | 0.000^**^ |  | 0.121 | 0.008^**^ |  | 0.233 | 0.000^**^ |
| Indecisiveness | 0.251 | 0.000^**^ |  | 0.254 | 0.000^**^ |  | 0.247 | 0.000^**^ |
| Thinking retardation | 0.195 | 0.000^**^ |  | 0.216 | 0.000^**^ |  | 0.180 | 0.000^**^ |
| Social withdrawal | 0.166 | 0.000^**^ |  | 0.179 | 0.000^**^ |  | 0.155 | 0.000^**^ |
| Poor interpersonal relationships | 0.273 | 0.000^**^ |  | 0.288 | 0.000^**^ |  | 0.259 | 0.000^**^ |
| Impaired social and professional functions | 0.196 | 0.000^**^ |  | 0.199 | 0.000^**^ |  | 0.189 | 0.000^**^ |

Note: ^*^ *P* < 0.05, ^**^ *P* < 0.01
